# Draft Genome Sequences of 25 Mycobacterium marinum Strains Isolated from Animals and Environmental Components in Aquaria and an Aquaculture Farm

**DOI:** 10.1128/mra.00851-22

**Published:** 2022-09-26

**Authors:** Takeshi Komine, Hanako Fukano, Mari Inohana, Yoshihiko Hoshino, Osamu Kurata, Shinpei Wada

**Affiliations:** a Laboratory of Aquatic Medicine, School of Veterinary Medicine, Nippon Veterinary and Life Science University, Musashino, Tokyo, Japan; b Department of Mycobacteriology, Leprosy Research Center, National Institute of Infectious Diseases, Higashi-Murayama, Tokyo, Japan; University of Delaware

## Abstract

Mycobacterium marinum is a ubiquitous nontuberculous mycobacterium that causes infections in various animals. Here, we report the annotated draft genome sequences of 25 strains isolated from vertebrates, invertebrates, and environmental components in aquaria and an aquaculture farm in Japan, sampled between 2015 and 2020.

## ANNOUNCEMENT

Mycobacterium marinum is a nontuberculous mycobacterium (NTM) and zoonotic pathogen that has been isolated from mammals, fish, amphibians, reptiles, birds, invertebrates, and protists (1–8); M. marinum is also widely distributed in nature, especially in aquatic environments (9–13). We sequenced the genomes of 25 strains of M. marinum that had been isolated from vertebrates, invertebrates, and environmental components in aquaria and an aquaculture farm in Japan between 2015 and 2020.

Bacterial isolations from the samples were conducted on two different culture media, i.e., Middlebrook 7H11 agar supplemented with 10% BBL Middlebrook oleic acid-albumin-dextrose-catalase (OADC) enrichment (Becton, Dickinson and Company) and 0.5% Tween 80 or slants of 2% Ogawa egg medium (Kyokuto Pharmaceutical Industrial Co., Ltd.), following chemical decontamination with *N*-acetyl-l-cysteine–sodium citrate NaOH, 4% NaOH, or 1 N HCl. Isolates were confirmed to be M. marinum through phenotypic and molecular examinations, as reported previously (14).

Frozen stocks (−80°C in 20% glycerol) of M. marinum strains were streaked onto the Ogawa egg medium slants, and single colonies were grown at 25°C for approximately 2 weeks. Bacterial pellets were boiled at 95°C for 15 min, frozen at −20°C overnight, and crushed twice (4,500 rpm for 1 min) by using MicroSmash (TOMY Digital Biology Co., Ltd., Japan), and then genomic DNA was extracted using the NucleoSpin Plant II kit (Macherey-Nagel GmbH & Co. KG) according to the manufacturer’s instructions. Sequencing libraries were prepared using the Nextera XT DNA library preparation kit (Illumina). The libraries were sequenced on the Illumina HiSeq X system (2 × 150 bp), as performed by Macrogen Japan Corp. The quality of raw reads was assessed with FastQC v0.11.9 (15). The sequence reads were trimmed for quality using fastp v0.20.1 (16) and assembled *de novo* using Platanus_B v1.1.0 (17). Genome completeness and contamination were assessed using CheckM v1.0.7 (18), and automated annotation was conducted with the DNA Data Bank of Japan (DDBJ) Fast Annotation and Submission Tool (DFAST) (https://dfast.nig.ac.jp). The annotated assemblies for all 25 strains were deposited in the DDBJ. Average nucleotide identity (ANI) values between the query and genomes of the reference strains, namely, Mycobacterium pseudoshottsii JCM 15466 (GenBank accession number AP018410.1), Mycobacterium shottsii JCM 12657 (GenBank accession number AP022572.1), Mycobacterium liflandii 128FXT (GenBank accession number CP003899.1), Mycobacterium ulcerans Agy99 (GenBank accession number CP000325.1), M. ulcerans SGL03 (GenBank accession number LR135168.1), M. marinum MMA1 (GenBank accession number CP058277.1), M. marinum M (GenBank accession number NC_010612.1), M. marinum E11 (GenBank accession number HG917972.2), M. marinum CCUG 20998 (GenBank accession number CP024190.1), and M. marinum ATCC 927 (GenBank accession number AP018496.1), from the National Center for Biotechnology Information (NCBI) were calculated using FastANI Galaxy v1.3 (https://usegalaxy.eu/root?tool_id=toolshed.g2.bx.psu.edu/repos/iuc/fastani/fastani/1.3). Default parameters were used except where otherwise noted.

The 25 strains were closest to M. marinum, with ANI values of 98.8% to 99.6%. The draft genomes were found to be 97.0% to 99.5% complete and to contain 0.5% to 3.1% contamination. All other genomic statistics are given in Table 1.

**TABLE 1:** Information on the 25 isolated strains of Mycobacterium marinum and genome assembly statistics

Strain	Isolate source	Year of isolation	Location of isolation	No. of raw reads	Genome size (bp)	No. of contigs	*N*_50_ (bp)	Coverage (×)	Total no. of coding sequences	G+C content (%)	DRA accession no.	GenBank accession no. for contigs
NJB1507	Bullseye (Parapriacanthus ransonneti)	2015	Fukushima, Japan	3,428,728	6,259,521	535	51,386	72	5,189	65.5	DRR337890	BQLB01000001–BQLB01000535
NJB1604	Ayu (Plecoglossus altivelis)	2016	Kanagawa, Japan	2,602,254	6,509,590	597	42,515	54	5,442	65.5	DRR337891	BQLC01000001–BQLC01000597
NJB1728-216S	Sharphead flyingfish (Hirundichthys oxycephalus)	2018	Shimane, Japan	4,158,042	5,934,806	407	33,854	84	5,132	65.6	DRR337892	BQLD01000001–BQLD01000407
NJB1728-910S	Sharphead flyingfish (Hirundichthys oxycephalus)	2018	Shimane, Japan	2,925,916	5,946,452	418	33,697	58	5,127	65.6	DRR337893	BQLE01000001–BQLE01000418
NJB1728-e18	Filter sand in aquarium tank	2019	Shimane, Japan	4,124,536	5,915,155	379	35,950	86	5,117	65.7	DRR337894	BQLH01000001–BQLH01000379
NJB1728-e22	Filter sand in aquarium tank	2020	Shimane, Japan	2,544,976	5,943,733	400	33,735	56	5,117	65.6	DRR337895	BQLF01000001–BQLF01000400
NJB1728-e24	Filter sand in aquarium tank	2020	Shimane, Japan	2,957,512	5,966,832	371	37,263	66	5,160	65.7	DRR337896	BQLI01000001–BQLI01000371
NJB1728-f10	Japanese flyingfish (Cypselurus hiraii)	2019	Shimane, Japan	1,776,280	5,927,986	444	32,203	35	5,108	65.6	DRR337897	BQLJ01000001–BQLJ01000444
NJB1728-f31	Sharphead flyingfish (Hirundichthys oxycephalus)	2019	Shimane, Japan	2,396,202	5,962,589	446	35,714	49	5,133	65.7	DRR337898	BQLK01000001–BQLK01000446
NJB1800-1	Slender bitterling (Acheilognathus yamatsutae)	2018	Niigata, Japan	3,186,550	6,003,274	350	45,188	71	5,171	65.7	DRR337899	BQLL01000001–BQLL01000350
NJB1808	Slender wrasse (Suezichthys gracilis)	2018	Shimane, Japan	4,651,326	5,906,789	392	36,645	94	5,112	65.6	DRR337900	BQLM01000001–BQLM01000392
NJB1808-e29	Bottom sand in aquarium tank	2020	Shimane, Japan	3,441,772	5,920,818	399	33,771	76	5,112	65.7	DRR337901	BQLG01000001–BQLG01000399
NJB1809-1	Pantropical spotted dolphin (Stenella attenuata)	2018	Okinawa, Japan	2,773,968	6,405,613	433	57,061	62	5,347	65.7	DRR337902	BQLN01000001–BQLN01000433
NJB1907-E11	Eelgrass (Zostera marina)	2019	Tokyo, Japan	3,544,950	5,978,197	382	36,430	78	5,158	65.7	DRR337903	BQLO01000001–BQLO01000382
NJB1907-E19	Rearing water of aquarium tank	2020	Tokyo, Japan	3,153,280	5,975,450	445	32,825	70	5,128	65.7	DRR337904	BQLP01000001–BQLP01000445
NJB1907-E39	Bottom sand in aquarium tank	2020	Tokyo, Japan	4,062,840	5,975,388	437	35,278	90	5,135	65.7	DRR337905	BQLQ01000001–BQLQ01000437
NJB1907-E49	Eelgrass (Zostera marina)	2020	Tokyo, Japan	4,364,488	5,972,808	435	37,449	97	5,139	65.6	DRR337906	BQLR01000001–BQLR01000435
NJB1907-E78	Sea cucumber (Apostichopus japonicus)	2020	Tokyo, Japan	2,395,764	5,969,857	396	36,326	53	5,147	65.7	DRR337907	BQLS01000001–BQLS01000396
NJB1907-E8	Bottom sand in aquarium tank	2019	Tokyo, Japan	1,206,776	5,964,802	544	30,062	26	5,107	65.6	DRR337908	BQLT01000001–BQLT01000544
NJB1907-E90	Ragworm (*Marphysa* sp.)	2020	Tokyo, Japan	1,703,520	5,952,250	447	31,681	37	5,138	65.6	DRR337909	BQLU01000001–BQLU01000447
NJB1907-f22	Pygmy filefish (Rudarius ercodes)	2019	Tokyo, Japan	1,734,322	5,963,590	465	30,823	38	5,136	65.6	DRR337910	BQLV01000001–BQLV01000465
NJB1907-f3	Pygmy filefish (Rudarius ercodes)	2019	Tokyo, Japan	2,459,240	5,968,709	429	33,713	54	5,149	65.6	DRR337911	BQLW01000001–BQLW01000429
NJB1907-f34a	Grey mullet (Mugil cephalus)	2020	Tokyo, Japan	1,514,586	5,946,765	484	31,499	33	5,091	65.7	DRR337912	BQLX01000001–BQLX01000484
NJB1907-f34b	Grey mullet (Mugil cephalus)	2020	Tokyo, Japan	4,077,620	5,957,620	418	33,713	91	5,132	65.7	DRR337913	BQLA01000001–BQLA01000418
NJB1907-f44	Pygmy filefish (Rudarius ercodes)	2020	Tokyo, Japan	3,568,982	5,979,843	421	35,323	79	5,141	65.7	DRR337914	BQLY01000001–BQLY01000421

### Data availability.

The genome sequencing and assembly projects have been deposited in the DDBJ under BioProject accession number PRJDB12467. See Table 1 for the DDBJ Sequence Read Archive (DRA) and DDBJ accession numbers.
